# Osteosarcoma

**DOI:** 10.1054/bjoc.2001.1770

**Published:** 2001-05

**Authors:** T Philip, J Y Blay, M Brunat-Mentigny, C Carrie, P Chauvot, F Farsi, B Fervers, J C Gentet, F Giammarile, R Kohler, S Mathoulin, L M Patricot, P Thiesse

**Affiliations:** Centre Léon Bérard, Lyon; Cancer Research Campaign, Paris; CHU, La Timone, Marseille; CHU, Hôpital Edouard-Herriot, Lyon; Institut Bergonié, Bordeaux; CHU, Hôpital Croix-Rousse, Lyon, France


					Osteosarcoma is the most common malignant bone tumour found
in children and young adults. The annual incidence is 65 cases per
year which represents 5% of all childhood cancers (0.5 cases per
100 000 per year). Non-metastatic osteosarcoma is currently
curable in about 70% of cases.
These guidelines do not relate to radiation-induced osteosar-
coma or that occurring in Paget's disease. They were validated in
December 1998 and will be updated according to the publication
of new data.
DIAGNOSIS
Plain X-rays of bone remain indispensable for confirming the
presence of bone pathology. Lesions are typically localized in the
metaphyseal region of long bones, as mixed osteolytic osteoblastic
lesions or cortical lytic lesions, with periosteal reaction and
adjacent soft-tissue mass.
Technetium bone scanning is used to detect the growth plates,
and also skip metastases and distant spread. The examination
should be done in three phases to study the functional characteris-
tics of the radio-isotope uptake over time. A thoracic CT scan
detects lung metastases not visible on chest X-ray. Thin (about 1
cm) cuts should be taken, if possible by spiral scanning (level of
evidence B). MRI and bone scan must be done before surgical
biopsy to avoid artefacts from haemorrhage, oedema and bone
healing. CT scanning and arteriography are no longer used
routinely to study the primary tumour. An arteriogram done imme-
diately preoperatively can in some cases identify venous tumour
emboli, provided this is a dynamic scan.
DIAGNOSIS
The biopsy must be taken by the surgeon who is to eventually do
the definitive surgery. He/she must be experienced in this type of
surgery. The biopsy should be of adequate size and representative
of the tumour. Surgical drainage should be avoided. The incision
must be placed in an area that will be excised at the time of even-
tual resection. In some very specialized units, a core-needle biopsy
may be sufficient to make a firm diagnosis, but this requires partic-
ular expertise with regard to interpretation and is not yet standard
practice. The role of the pathologist is very important for both the
diagnosis and in evaluating the subsequent response to chemo-
therapy (level of evidence A).
STAGING
Patients are generally classified as having metastatic or non-meta-
static disease. Age, tumour volume and site of tumour have been
reported as prognostic variables, but these are not consistent.
Alkaline phosphatase, LDH, renal function and cardiac echogram
should be documented prior to therapy.
TREATMENT MODALITIES
Children should be treated according to an appropriate multicentre
trial, such as SFOP and adults within FNCLCC or EORTC protocols.
Chemotherapy
Protocols combining neo-adjuvant chemotherapy and adjuvant
chemotherapy have superior efficacy to protocols of adjuvant
chemotherapy alone (level of evidence B). However, there are
no randomized trials comparing these therapeutic options.
Chemotherapy must be given by teams experienced in the use of
aggressive and toxic protocols with provision of full medical
and haematological supportive care. Preoperative chemotherapy
generally combines a number of agents (standard). A variety of
protocols can be proposed: high-dose methotrexate + doxorubicin,
doxorubicin + cisplatin, high-dose methotrexate + cisplatin +
doxorubicin, ifosfamide + cisplatin (Figure 1).
High-dose methotrexate is effective and widely used in children
(level of evidence B). It is generally given at a dose of at least
12 g m-2
to children. In adults, a test dose may be used to define
the dose that will give an identical area under the concentration
curve for all patients, and is usually at least 8 g m-2
. In general, the
scheduling of methotrexate is identical in adults to children. It
must be possible to measure methotrexate levels and to support
with dialysis if necessary. The methods of high-dose methotrexate
administration will vary according to different protocols, but facil-
ities to provide rigorous hydration, clinical surveillance, regular
blood tests and folinic acid rescue must be in place at all times.
Randomized trials have suggested a combination of cisplatin
and doxorubicin may be comparable to more complex multiagent
regimens (level of evidence B).
Surgery
Surgery must be undertaken by an experienced surgeon who is
familiar with the indications for amputation if conservative
surgery is contraindicated. For localized disease, the biopsy scar
should be resected and the tumour removed `en bloc' without
being opened (level of evidence B) (Figure 2). The limits of
excision must be wide and clear histologically. A sample of the
proximal marrow must be taken for analysis. Several surgical
options are possible: reconstructive surgery using prostheses,
reconstruction using bone graft, either autologous or allogeneic,
and rotationplasty.
Osteosarcoma
T Philip1
, JY Blay1
, M Brunat-Mentigny1
, C Carrie1
, P Chauvot1
, F Farsi1
, B Fervers1,2
, JC Gentet3
, F Giammarile1
,
R Kohler4
, S Mathoulin5
, LM Patricot6
and P Thiesse1
1
Centre Leon Berard, Lyon; 2
FNCLCC, Paris; 3
CHU, La Timone, Marseille; 4
CHU, Hopital Edouard-Herriot, Lyon; 5
Institut Bergonie, Bordeaux; 6
CHU, Hopital
Croix-Rousse, Lyon, France
78
British Journal of Cancer (2001) 84(Supplement 2), 78-80
(c) 2001 FNCLCC
doi: 10.1054/ bjoc.2001.1770, available online at http://www.idealibrary.com on
Osteosarcoma 79
British Journal of Cancer (2001) 84 (Supplement 2), 78-80
(c) 2001 FNCLCC
The result of primary chemotherapy is documented by Huvos'
histological grading on excised tumour (Figure 3). Good and bad
responders to chemotherapy are defined. At present in France, the
definition of a good response is if there is less than 10% of viable
tumour cells in the sample. The response to preoperative chemo-
therapy is the single most important prognostic factor (level of
evidence B). Various medical imaging techniques are under evalu-
ation to determine the efficacy of chemotherapy. These include
MRI, PET and Thallium scans. The results are at present unreli-
able in predicting response (level of evidence A). These tests
should only be done in the context of an evaluable protocol.
The existence of metastases at presentation, in particular if they
are single, should not lead to abandoning the possibility of curative
treatment (level of evidence B). Amputation of limbs is only
done under exceptional circumstances. Aggressive surgery to lung
metastases following primary chemotherapy may be appropriate
(Figure 4).
Radiotherapy
Radiotherapy may be indicated in the case of inoperable tumour.
Options for irradiation include high dose, between 55-70 Gy
according to site, and also photon or neutron therapy. Radiotherapy
may also be useful for palliation of locally recurrent disease.
FOLLOW-UP
Few studies have compared the method and timing of follow up
for osteosarcoma. Recommendations are as follows (level of
evidence B):
* Chest X-ray every 2 months, for years 1 and 2, every 3 months
for years 3 and 4, every 6 months for years 5 and 6, then
annually.
* CT scan (if there were lung metastases at presentation) every 6
months for the first year and then every year until the fourth
year.
* Plain X-ray of bone and MRI are only done in the case of
local symptoms. Technetium bone scan may be done every
4 months for years 1 and 2, every 6 months for years 3
and 4 and then only in the case of symptoms. The role of
bone scan remains debatable, however, as there is no
useful therapeutic intervention in the event of distant bone
metastases.
INTERNAL REVIEWERS
BN Bui (Institut Bergonie, Bordeaux), MC Demaille (Centre
Oscar Lambret, Lille), C. Kalifa (Institut Gustave Roussy,
Villejuif), P. Pouillart (Institut Curie, Paris), J. Stines (Centre
Alexis Vautrin, Nancy) and JM Zucker (Institut Curie, Paris).
Localized and metastatic osteosarcoma:
preoperative chemotherapy
Standard
combination chemotherapy
Options
* high-dose methotrexate + doxorubicin
* adriamycin + cisplatin
* high-dose methotrexate + cisplatin
* ifosfamide + cisplatin
Recommendations
inclusion in a study protocol:
* adults: FNCLCC, EORTC
* children: SFOP, protocols containing high-dose methotrexate
no yes
Metastases at presentation?
Non-metastatic disease:
local treatment
(see Figure 2)
Metastatic disease:
local treatment
(see Figure 4)
Figure 1 Preoperative chemotherapy for osteosarcoma
Non-metastatic disease:
local treatment
Presurgical post-chemotherapy evaluation:
reassessment using the same modalities as at presentation
Contra-indication
to surgery?
no yes
Contra-indication to
conservative surgery?
no yes
Recommendations
* limb-conserving surgery with extended
excision and resection of tumour en bloc
* resection of biopsy scar
* reconstruction with prosthesis or bone
graft if required
Recommendation
amputation
Radiotherapy
Recommendation
photon or neutron therapy
Postoperative
chemotherapy
Postoperative
chemotherapy
Figure 2 Treatment of local disease
Metastatic and non metastatic disease:
post operative treatment
Good responder
Huvos grade III or IV in both
primary tumour and metastases?
Standard
postoperative chemotherapy
using the same regimen as
preoperatively
Standard
postoperative chemotherapy using alternative
agents active in osteosarcoma
Options
if methotrexate and doxorubicin given peroperatively
* ifosfamide + VP 16
* ifosfamide + cisplatin
Recommendation
evaluable study protocol
* adults: FNCLCC, EORTC
* children: SFOP
* adults and children: phase II studies
Follow-up Follow-up
yes no
Figure 3 Postoperative treatment
80 T Philip et al
British Journal of Cancer (2001) 84 (Supplement 2), 78-80 (c) 2001 FNCLCC
EXTERNAL REVIEWERS
JH Aubriot (CHU, Caen), G Bollini (Hopital Timone, Marseille),
H Carlioz (Hopital Trousseau, Paris), P Dalivoust (Clinique
Residence du Parc, Marseille), J Dubousset (Hopital Saint-
Vincent-de-Paul, Paris), P Guichard (Polyclinique Bordeaux Nord,
Bordeaux), JJ Mallet (Faculte de la Medecine, Grange Blanche,
Lyon), Y Matillon (ANDEM, Paris), A Mazabraud (Cabinet
d'anatomie et cytologie pathologique, Bievres), C Raybaud
(Hopital des Enfants, Marseille), D Sommelet (CHU, Vandoeuvre-
Les-Nancy), P Tramond (Hopital R Boulin, Libourne) and M
Urbajtel (Hopital d'Argenteuil, Argenteuil).
Figure 4 Treatment of metastatic disease
Metastatic disease: local treatment
Presurgical assessment
reevaluation using the same
modalities as at presentation
Standard
surgery to primary tumour and metastases
Recommendations
* limb-conserving surgery with extended excision and
en bloc resection
- excision of biopsy scar
- amputation only under exceptional circumstances
* metastatectomy according to site
Postoperative
chemotherapy

				

